# Measuring Patient Satisfaction: A Case Study to Improve Quality of Care at Public Health Facilities

**DOI:** 10.4103/0970-0218.62554

**Published:** 2010-01

**Authors:** Prahlad Rai Sodani, Rajeev K Kumar, Jayati Srivastava, Laxman Sharma

**Affiliations:** Department of Research, Institute of Health Management Research, Jaipur, India; 1 Directorate of Medical, Health and Family Welfare Services, Government of Rajasthan, Jaipur, India

**Keywords:** Patient satisfaction, public health facilities, quality of care

## Abstract

**Objective:**

The main objective of the study is to measure the satisfaction of OPD (Outpatient Department) patients in public health facilities of Madhya Pradesh in India.

**Materials and Methods:**

Data were collected from OPD patients through pre-structured questionnaires at public health facilities in the sampled eight districts of Madhya Pradesh. The data were analyzed using SPSS.

**Settings:**

Outpatient Departments of district hospital, civil hospital, community health centre, and primary health centre of the eight selected districts of Madhya Pradesh.

**Results:**

A total of 561 OPD patients were included in the study to know their perceptions towards the public health facilities, choosing health facility, registration process, basic amenities, perception towards doctors and other staff, perception towards pharmacy and dressing room services. It was found that most of the respondents were youth and having low level of education. The major reason of choosing the public health facility was inexpensiveness, infrastructure, and proximity of health facility. Measuring patient satisfaction were more satisfied with the basic amenities at higher health facilities compared to lower level facilities. It was also observed that the patients were more satisfied with the behavior of doctors and staff at lower health facilities compared to higher level facilities.

## Introduction

Patient satisfaction is one of the important goals of any health system, but it is difficult to measure the satisfaction and gauze responsiveness of health systems as not only the clinical but also the non-clinical outcomes of care do influence the customer satisfaction.(1) Patients' perceptions about health care systems seem to have been largely ignored by health care managers in developing countries. Patient satisfaction depends up on many factors such as: Quality of clinical services provided, availability of medicine, behavior of doctors and other health staff, cost of services, hospital infrastructure, physical comfort, emotional support, and respect for patient preferences.(2) Mismatch between patient expectation and the service received is related to decreased satisfaction.(3) Therefore, assessing patient perspectives gives them a voice, which can make public health services more responsive to people's needs and expectations.(4,5)

In the recent past, studies on patient satisfaction gained popularity and usefulness as it provides the chance to health care providers and mangers to improve the services in the public health facilities. Patients' feedback is necessary to identify problems that need to be resolved in improving the health services. Even if they still do not use this information systematically to improve care delivery and services, this type of feedback triggers a real interest that can lead to a change in their culture and in their perception of patients.(6)

The present paper is based on a comprehensive study conducted at public health facilities in the State of Madhya Pradesh to measure patient satisfaction who have availed services at outdoor patient department, in-door patient department and diagnostics/investigative. The main objective of this paper is to share the findings on patients' satisfaction about various components of out-door patient department (OPD) services. In this study, the OPD is defined as the hospital's department where patients received diagnoses and/or treatment but did not stay overnight.

## Materials and Methods

The state is divided into eight administrative divisions. To have a representative sample of the State, one district has been identified from each of the division. The identified districts were: Vidisha, Morena, Gwalior, Indore, Jabalpur, Sidhi, Sagar and Ujjain. To select OPD patients from each district, a sample of OPD patients were drawn from the public health facilities, i.e., district hospital (DH), civil hospital (CH), community health centre (CHC), and primary health centre (PHC). From each of the selected district, one DH, one CH, one CHC and one PHC were identified. Thus, in all, 561 OPD patients were covered from 32 public health facilities of the state [Table 1].

**Table 1:** Distribution of OPD patients according to type of public health facilities (in MP)

Sample units	DH	CH	CHC	PHC	Total
No. of facilities	8	8	8	8	32
No. of patients	244	157	80	80	561

DH - District hospital, CH - Civil hospital, CHC - Community health centre, PHC - Primary health centre

To carry out the proper scientific study, a set of well structured questionnaire containing close-ended questions was developed. The questionnaire was pre-tested. The finalized questionnaire was translated into Hindi, the State language, for administering purposes. The questionnaire covered the information related to patient's socio-economic characteristics, patient's choice of health facility, registration process, perception towards availability of basic amenities, behavior of doctors and other staff, facilities available in pharmacy and dressing room. Data were collected with the help of trained field investigators during the months of September and October 2007. The state government facilitated data collection from various facilities. After collecting the data from various health facilities, data editing and cleaning were done before the data entry. The data were analyzed using the SPSS version 12.

## Results and Discussions

### Characteristics of the OPD patients

It includes information on sex, age, and literacy level of the OPD patients. Table 2 shows that 47% patients were males and rest 53% were females. The maximum number of respondents (45%) belongs to the age group of 16-30 years and minimum respondents (6%) to 0-15 year age group. The education level of the respondents was very poor as most of them were either illiterate (39%) or primary passed (18%).

**Table 2:** Sex, age and education level of OPD patients at public health facilities in MP

	DH n = 244 (%)	CH n = 157 (%)	CHC n = 80 (%)	PHC n = 80 (%)	Total n = 561 (%)
Sex					
Male	118 (48)	61 (39)	40 (50)	43 (54)	262 (47)
Female	126 (52)	96 (61)	40 (50)	37 (46)	299 (53)
Age group (Yrs)					
0-15	10 (4)	15 (9)	7 (9)	3 (4)	35 (6)
16-30	114 (47)	74 (47)	34 (42)	30 (37)	252 (45)
31-50	77 (31)	48 (31)	20 (25)	30 (37)	175 (31)
50 and above	43 (18)	20 (13)	19 (24)	17 (21)	99 (18)
Education level					
Illiterate	93 (38)	57 (36)	40 (50)	30 (37)	220 (39)
Primary	40 (16)	39 (25)	11 (14)	13 (16)	103 (18)
Middle	36 (15)	12 (8)	14 (17)	18 (22)	80 (14)
Secondary	25 (10)	24 (15)	6 (7)	10 (12)	65 (12)
High secondary	32 (13)	17 (11)	7 (9)	8 (10)	64 (11)
Graduated above	18 (7)	8 (5)	2 (2)	1 (1)	29 (5)

DH - District hospital, CH - Civil hospital, CHC - Community health centre, PHC - Primary health centre

### Reasons for choosing the facility

Inexpensiveness and good infrastructure was one of the most cited reasons (83%) for choosing the public health facilities by the OPD patients. Majority of the patients (81%) said that the main source of information about the hospital was family members/relatives. More than half of the respondents reached the hospital on foot. The time taken to reach the hospital was less than 15 minutes for more than half of the respondents (58%). Almost all the respondents did not find any problem in locating the hospital (93%) or locating different departments within the hospital (87%). One of the major reasons for choosing the public health facility was unavailability of other health facilities in the area.

### Registration process

Regarding the registration process, 64% OPD patients said the registration counter was over crowded. It was more observed at the higher level health facility (DH) compared with lower level health facilities (PHC). However, the patients were happy with the behavior of registration clerk at all the facilities [Table 3].

**Table 3:** Number of visits in last one year and reasons for selecting the public health facilities in MP

Variables	DH n = 244 (%)	CH n = 157 (%)	CHC n = 80 (%)	PHC n = 80 (%)	Total n = 561 (%)
Reasons for selecting the facility					
In-expensive/good infrastructure	206 (84)	127 (81)	69 (86)	61 (76)	463 (83)
Skilled doctors/nurse	38 (16)	30 (19)	11 (14)	19 (24)	98 (18)
Source of information					
Neighbor	43 (18)	41 (26)	17 (21)	8 (10)	109 (19)
Family members/relatives	201 (82)	116 (74)	63 (79)	72 (90)	452 (81)
Mode of transportation					
On foot	86 (35)	88 (56)	43 (54)	74 (95)	291 (52)
Rickshaw/tanga	49 (20)	10 (6)	8 (10)	5 (6)	72 (13)
Bus/car/tractor/truck	65 (27)	24 (16)	16 (20)	0 (0)	105 (19)
Cycle/auto/motorcycle	44 (18)	35 (22)	13 (16)	1 (1)	93 (17)
Transportation cost (in Rs.)					
Free of cost	103 (42)	97 (62)	53 (66)	74 (93)	327 (58)
1-10	65 (27)	26 (16)	12 (15)	5 (6)	108 (19)
11-50 and above	76 (31)	34 (22)	15 (19)	1 (1)	126 (22)
Time taken					
1-15	128 (52)	81 (52)	44 (55)	73 (92)	326 (58)
16-30	52 (21)	36 (23)	15 (19)	5 (6)	108 (19)
31 and above	64 (26)	40 (25)	21 (26)	2 (2)	127 (23)
Problem in locating the hospital					
Yes	25 (10)	6 (4)	4 (5)	2 (2)	37 (7)
No	219 (90)	151 (96)	76 (95)	78 (98)	524 (93)
Problem in locating different department within the hospital					
Yes	55 (23)	9 (6)	3 (4)	4 (5)	71 (13)
No	189 (77)	148 (94)	77 (96)	76 (95)	490 (87)
Any other hospital nearer to your house					
Yes	61 (25)	16 (10)	2 (2)	0 (0)	79 (14)
No	183 (75)	141 (90)	78 (98)	80 (100)	482 (86)
Was registration counter overcrowded					
Yes	161 (66)	113 (72)	57 (71)	27 (34)	358 (64)
No	83 (34)	44 (28)	23 (29)	53 (66)	203 (36)
Behavior of the registration clerk					
Good	237 (97)	153 (97)	79 (99)	74 (93)	543 (97)
Poor	7 (3)	4 (3)	1 (1)	6 (7)	18 (3)

DH - District hospital, CH - Civil hospital, CHC - Community health centre, PHC - Primary health centre

### Basic amenities

It was observed that respondents were more satisfied with the basic amenities such as seating arrangement for the patients and attendants, cleanliness, fans, toilets, drinking water, and telephone facility at higher level facility i.e. district and civil hospitals as compared to community health center and primary health center. The main reason being the higher level facilities have better infrastructure than the lower health facilities.

Table 4 shows the perception of OPD patients on availability of basic amenities in the studied public health facilities. More than half of the respondents (54%) found sitting arrangement adequate and 71% respondents found hospitals adequately cleaned. Most of the OPD patients were happy with the lighting arrangements at the higher facilities. Regarding toilet facility, 44% respondents said it was available and clean, but 49% respondents said it was available but dirty. More than half of the respondents (69%) reported that the drinking water facility was available while 56% of the respondents reported lack of telephone facility in hospitals.

**Table 4:** Client perception on basic amenities at public health facilities according to selected attributes

Selected attributes	DH n = 244 (%)	CH n = 157 (%)	CHC n = 80 (%)	PHC n = 80 (%)	Total n = 561 (%)
Sitting arrangement for the patients and attendants					
Adequate	114 (47)	122 (78)	31 (39)	35 (44)	302 (54)
Inadequate	130 (53)	35 (22)	49 (61)	45 (56)	259 (46)
Cleanliness					
Adequate	158 (65)	138 (88)	59 (74)	41 (51)	396 (71)
Inadequate	86 (35)	19 (12)	21 (26)	39 (49)	165 (29)
Lighting arrangement					
Good	123 (50)	124 (79)	44 (55)	43 (54)	334 (60)
Satisfactory	106 (44)	30 (19)	32 (40)	25 (31)	193 (34)
Poor	15 (6)	3 (2)	4 (5)	12 (15)	34 (6)
Fans					
Adequate	116 (48)	124 (79)	45 (56)	19 (24)	304 (54)
Inadequate	128 (52)	33 (21)	35 (44)	61 (76)	257 (46)
Toilets					
Available and clean	84 (34)	106 (68)	32 (40)	27 (34)	249 (45)
Available but dirty	153 (63)	50 (31)	37 (46)	35 (44)	275 (49)
Unavailable	7 (3)	1 (0.6)	11 (14)	18 (22)	37 (7)
Drinking water					
Available	199 (82)	128 (82)	47 (59)	13 (16)	387 (69)
Not available	45 (18)	29 (18)	33 (41)	67 (84)	174 (31)
Telephone					
Available	119 (49)	86 (55)	30 (37)	11 (14)	246 (44)
Not available	125 (51)	71 (45)	50 (63)	69 (96)	315 (56)

DH - District hospital, CH - Civil hospital, CHC - Community health centre, PHC - Primary health centre

### Perception of OPD patients towards doctors

It was observed from the data that the waiting time for OPD patients at the higher level health facilities is more than the lower level health facilities, because of the high patient load at district and civil hospitals. Most of the patients (78%) at PHC said that they have to wait less than 10 minutes for the doctor whereas in case of DH, CH and CHC; 54%, 52%, and 51% patients respectively said so. Majority of the patients (above 85%) have observed that doctor's behavior was good at all the facilities and they also felt that the doctor has given adequate time to see the patients [Table 5].

**Table 5:** Client perception on doctor's service at public health facilities in MP

Doctor's service	DH n = 244 (%)	CH n = 157 (%)	CHC n = 80 (%)	PHC n = 80 (%)	Total n = 561 (%)
Time taken by the doctor to attend the patient (in minutes)					
≤10	131 (54)	82 (52)	41 (51)	62 (78)	316 (56)
11 to 30	90 (37)	54 (34)	37 (46)	13 (16)	194 (35)
30 and above	23 (9)	21 (13)	2 (2)	5 (6)	51 (9)
Behavior of the doctor					
Good	218 (89)	145 (92)	78 (98)	69 (86)	510 (91)
Satisfactory	26 (11)	12 (8)	2 (2)	11 (14)	51 (9)
Adequate time given by the doctor					
Yes	231 (95)	147 (94)	80 (100)	74 (93)	532 (95)
No	13 (5)	10 (6)	0 (0)	6 (7)	29 (5)

DH - District hospital, CH - Civil hospital, CHC - Community health centre, PHC - Primary health centre

### Pharmacy

Out of 561 respondents, 433 had availed the pharmacy facility. Data revealed that the patients at lower level health facilities (CHC and PHC) were more satisfied with the queue system than at the higher level health facilities (DH and CH). Most of the OPD patients also perceived that the behavior of the pharmacist was good particularly at the higher level facilities (DH and CH). Behavior of pharmacist was either good or satisfactory for all the OPD patients. Regarding the quality of drugs, OPD patients were more happy at CHC and PHC as compared to DH and CH and the overall response to the quality of drugs was either good (64%) or satisfactory (33%) and only 3% considered it poor [Table 6].

**Table 6:** Client perception on pharmacy related services at public health facilities in MP

	DH n = 164 (%)	CH n = 128 (%)	CHC n = 72 (%)	PHC n = 69 (%)	Total n = 433 (%)
Discipline in queue system					
Good	119 (73)	85 (66)	66 (92)	54 (78)	324 (75)
Satisfactory	41 (25)	33 (26)	3 (4)	12 (17)	89 (20)
Poor	4 (2)	10 (8)	3 (4)	3 (5)	20 (5)
Behavior of the pharmacist					
Good	116 (71)	95 (74)	54 (75)	45 (65)	310 (72)
Satisfactory	48 (29)	33 (26)	18 (25)	24 (35)	123 (28)
Quality of drugs					
Good	106 (65)	75 (59)	49 (68)	49 (71)	279 (64)
Satisfactory	52 (32)	49 (38)	21 (29)	20 (29)	142 (33)
Poor	6 (3)	4 (3)	2 (3)	0 (0)	12 (3)

DH - District hospital, CH - Civil hospital, CHC - Community health centre, PHC - Primary health centre

### Dressing room

Out of 561, only 131 respondents utilized the dressing room facility. Of these, 63% patients felt that the dressing room was over crowded. The problem of over crowding in dressing rooms was felt more by the patients at DH (69%) and CH (72%) than the CHC (63%) and PHC (37%). However, majority of the respondents (92%) were satisfied with the promptness in providing service at dressing rooms at all the health facilities. In all, most of the patients (73%) found the cleanliness of dressing room good. The cleanliness of highest level facilities (DH) is better than the lowest level facilities (PHC). Majority (82%) of the patients perceived the behavior of the dressing room staff as good. However, at DH level, only 62% OPD patients considered the behavior of dressing room staff good [Table 7]. The reason might be over crowding of the patients and the heavy patient load at higher level facilities.

**Table 7:** Client perception on dressing room services at public health facilities in MP

	DH n = 45 (%)	CH n = 43 (%)	CHC n = 19 (%)	PHC n = 24 (%)	Total n = 131 (%)
Problem of overcrowding					
Yes	31 (69)	31 (72)	12 (63)	9 (37)	83 (63)
No	14 (31)	12 (28)	7 (37)	15 (63)	48 (37)
Promptness in services					
Yes	36 (80)	42 (98)	19 (100)	23 (96)	120 (92)
No	9 (20)	1 (2)	0 (0)	1 (4)	11 (8)
Cleanliness					
Good	28 (62)	38 (88)	17 (90)	12 (50)	95 (73)
Satisfactory	16 (36)	4 (10)	1 (5)	8 (33)	29 (22)
Poor	1 (2)	1 (2)	1 (5)	4 (17)	7 (5)
Behavior of the staff					
Good	28 (62)	40 (93)	19 (100)	20 (83)	107 (82)
Satisfactory	17 (28)	3 (7)	0 (0)	4 (17)	24 (18)

DH - District hospital, CH - Civil hospital, CHC - Community health centre, PHC - Primary health centre

## Conclusions

The study findings suggest that following measures may be taken by the policy makers and hospital administrators to increase the patient satisfaction at public health facilities: 1) Efforts should be made to reduce the patient load at the higher level facilities so that doctors and other staff can give more attention and time to the patients; 2) Efforts are also needed to strengthen infrastructure and human resources at the lower level health facilities. The findings of the present study can be utilized to improve the services at public health facilities of the state resulting in the more satisfaction of patients availing such public health facilities.

## References

[1] Agrawal D (2006). Health sector reforms: Relevance in India. Indian J Community Med.

[2] Jenkinson C, Coulter A, Bruster S, Richards N, Chandola T (2002). Patients' experiences and satisfaction with health care: Results of a questionnaire study of specific aspects of care. Qual Saf Health Care.

[3] McKinley RK, Roberts C (2001). Patient satisfaction with out of hours primary medical care. Qual Health Care.

[4] World Health Organization (2000). The World Health Report 2000-Health Systems: Improving Performance.

[5] Rao KD, Peters DH, Bandeen-Roche K (2006). Towards patient-centered health services in India- a scale to measure patient perceptions of quality. Int J Qual Health Care.

[6] Boyer L, Francois P, Doutre E, Weil G, Labarere J (2006). Perception and use of the results of patient satisfaction surveys by care providers in a French teaching hospital. Int J Qual Health Care.

